# The US framework for understanding, preventing, and caring for the mental health needs of service members who served in combat in Afghanistan and Iraq: a brief review of the issues and the research

**DOI:** 10.3402/ejpt.v5.24713

**Published:** 2014-08-14

**Authors:** Carl Andrew Castro

**Affiliations:** Center for Innovation and Research on Veterans and Military Families, School of Soical Work, University of Southern California, Los Angeles, CA, USA

**Keywords:** PTSD, combat, military

## Abstract

This paper reviews the psychological health research conducted in the United States in support of combat veterans from Iraq and Afghanistan, using the Military Psychological Health Research Continuum, which includes foundational science, epidemiology, etiology, prevention and screening, treatment, follow-up care, and services research. The review is limited to those studies involving combat veterans and military families. This review discusses perplexing issues regarding the impact of combat on the mental health of service members such as risk and resilience factors of mental health, biomarkers of posttraumatic stress syndrome (PTSD), mental health training, psychological screening, psychological debriefing, third location decompression, combat and suicide, the usefulness of psychotherapy and drug therapy for treating PTSD, role of advanced technology, telemedicine and virtual reality, methods to reduce stigma and barriers to care, and best approaches to the dissemination of evidence-based interventions. The mental health research of special populations such as women, National Guardsmen and reservists, and military families is also presented. The review concludes by identifying future areas of research.

There is no debate that combat adversely affects mental health. The demands of conducting combat operations day-in and day-out places a tremendous burden on the physical and psychological health of the service member. The effects of the demands of combat on the mental health of US service members have been documented for World War I, World War II, the Korean War, the Vietnam War, and the Gulf War (Ginzberg, 1959; Harris, Mayer, & Becker, 1955; Marshall, 1947; Perkins, 1955; Peterson & Chambers, 1952; Salmon, 1929; Stretch et al., 1996; Swank & Marchand, 1946). Since November 2001, the US military has been engaged in combat operations in Afghanistan and from March 2003 to August 2010 the United States has simultaneously been conducting combat operations in Iraq, resulting in service members enduring multiple, prolonged, deployments. Combined, the wars in Afghanistan and Iraq have resulted in more than 6,500 deaths and 50,000 wounded in action, with a further 118,000 receiving a diagnosis of posttraumatic stress syndrome (PTSD) after returning from deployment, yet still serving on active duty (Fischer, 2014). At the peak in 2012, more than 17,000 active duty combat veterans were being diagnosed with PTSD every year (Fischer, 2014). Of the more than 1.4 million combat veterans who have separated from military service since 2002, approximately 54% have received health care from the Veterans Administration (VA), with more than 404,000 combat veterans receiving a mental health diagnosis (VA, 2012).

As it became clear that the wars in Afghanistan and Iraq were taking an enormous toll on the mental health of the force (Hoge et al., 2004), in 2007, the United States vastly increased its medical research funding for mental health, totaling more than $1billion from 2007 to 2013. In order to ensure that the highest mental health research needs of the combat veteran were being addressed, the Department of Defense (DoD) developed the Psychological Health Research Continuum (Castro, 2013), which was subsequently adopted by the White House to guide interagency research strategy throughout the United States in the areas of PTSD, mTBI, and suicide research (White House, 2013).

This paper describes how the Psychological Health Research Continuum was used to guide the research conducted within the United States to ensure the best evidence-based prevention, treatment, and care would be provided to US combat veterans of the of the Afghanistan and Iraq wars. This paper also discusses the various issues regarding combat and mental health in which medical research can help provide answers and solutions. These issues include: risk and resilience factors of mental health, biomarkers of PTSD, mental health training, psychological screening, psychological debriefing, third location decompression (TLD), combat and suicide, the usefulness of psychotherapy and drug therapy for treating PTSD, role of advanced technology, telemedicine and virtual reality (VR), methods to reduce stigma and barriers to care, and best approaches to disseminate effective interventions. Finally, a brief review and discussion of the research looking at special populations, including National Guardsmen and reservists, female service members, ethnicity and race, and the military family is also provided. Without question not all the important issues regarding combat and mental health are addressed, yet the ones discussed here represent the central issues and concerns that the United States has been focused on over the last decade through medical research efforts. This review is also limited to those studies involving combat veterans (or their families); so important pre-clinical research findings are not discussed. Furthermore, given the volume of research that has emerged in the United States regarding the mental health and wellbeing of combat veterans, particular emphasis will be on research that has influenced or is likely to influence interventions. The paper concludes by identifying areas of research and issues that remain unresolved.

## Military occupational model for mental health

There are unique aspects of the military culture and context that need to be taken into consideration when developing and implementing intervention efforts involving active duty members, National Guardsmen and reservists, and military veterans. The military occupational mental health model (Adler & Castro, 2013) is a conceptual framework for understanding the relationship between occupationally relevant demands and subsequent mental health adjustment within the military. An occupational framework is valuable because it can enable a systematic approach to be undertaken in the care of its members. The military occupational mental health model integrates and extends several theoretical concepts of military health and wellness, most notably the Soldier Adaptation Model (Bliese & Castro, 2003), the stress buffering hypothesis (Karasek, 1979), theories of PTSD trauma (Foa et al., 2005; Resick et al., 2008), work-family conflict (Piftman, 1994), the greedy institution hypothesis (Segal, 1986), and various leadership theories, most notably the role of reciprocity and self-efficacy theory applied to relationship theories of leadership (Castro, Thomas, & Adler, 2006). The military occupational mental health model not only includes the relationships between standard work-related demands (such as work overload and role ambiguity), outcomes (health, attitude, and performance), and identifying the individual and organizational variables that might moderate those outcomes, such as leadership climate, collective efficacy, and policy decisions; it also emphasizes the relevance of personal and occupational background variables that occur prior to the stressor. Furthermore, the military occupational mental health model emphasizes the importance of occupational culture, prevention, and treatment within the military context.

Another central feature of the military occupational mental health model is the identification of the critical role that transitions play in affecting mental health. Transitions within the military (and life) can be major, and range from joining the military, getting married, deploying to combat, having children, returning home from combat, leaving the military, beginning a civilian career, and retirement. Just as important, yet often overlooked or minimized, are micro-transitions, which can also have major impacts on mental health. Within the military, micro-transitions might include moving from one duty station to another (although in some cases relocation can represent a major transition, especially if the move is overseas), going on and recovery from daily combat missions, preparing for and waiting on promotions, and having routine changes in unit leadership. The military occupational mental health model emphasizes that both the macro- and micro-transitions are important for sustaining the mental health and wellbeing of service members. Thus, the military occupational mental health model can be viewed as a transition-contextual theory.

## Psychological Health Research Continuum

**Fig 1:**
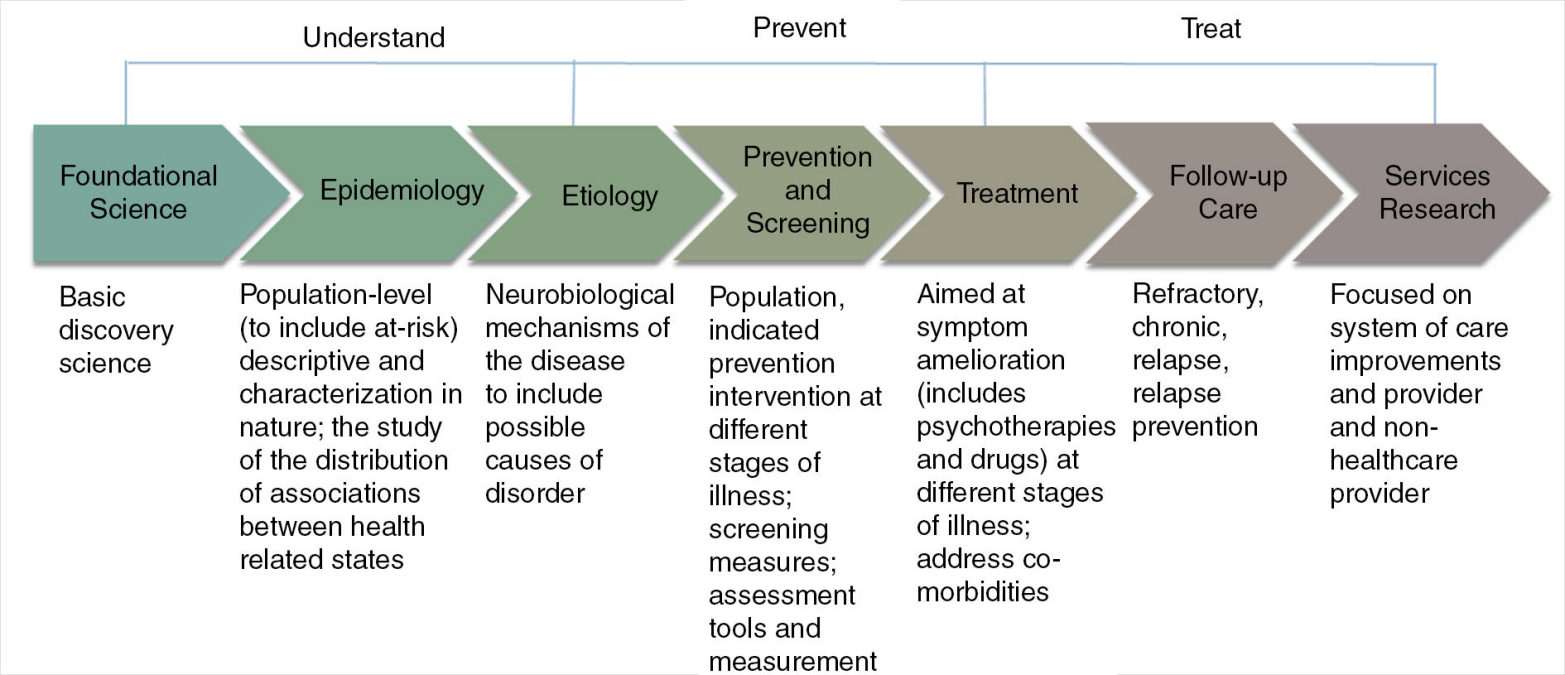
The military Psychological Health Research Continuum.

Figure 1 shows that the Psychological Health Research Continuum framework contains seven domains: foundational science, epidemiology, etiology, prevention and screening, treatment, follow-up care, and services research. For each of these topic areas, needs and possible solutions are identified from which research gaps can be identified. Based on a variety of factors, such as number of service members affected, severity of the medical condition, state of the science, and ongoing research, research gaps are prioritized for funding. This way, the United States can ensure that there is awareness of all identified needs, that all ongoing research within a particular research gap is integrated with other ongoing research, and that there is no unintended duplication of effort.

There are, however, several important areas that the Psychological Health Research Continuum framework does not capture. The Psychological Health Research Continuum does not 1) provide a projected timeline for when a solution for mental health care need will be provided, 2) identify how much more additional funding will be necessary to provide a solution, 3) describe the level of medical evidence that exists for all possible solutions, 4) consider the military context for a possible solution, or 5) include important subtopics of interest such as military veterans; National Guard and reservists; unique sex, ethnic and racial issues; and the complexities of comorbidities. Nevertheless, as long as these issues are kept in mind, the Psychological Health Research Continuum can be useful for organizing, determining funding priorities, and describing the extensive mental health research that is ongoing in the area of psychological health within the United States. The Psychological Health Research Continuum has been used to organize the research domains for many mental and behavioral health issues, including PTSD, suicide, alcohol and substance use, violence and anger within the military, risk-taking behaviors, and building resilience.

### Foundational science

Foundational science is basic discovery science. By definition, if the research involves the investigation of a mental health condition, no matter how reductionist, then the research is not foundational. Classic examples of foundational science topics include the structure of DNA, the origins of the universe, and how the brain encodes memories. Yet, foundational science is essential in order to understand the mechanisms and processes that underlie mental health disorders and diseases, which then can be the targets for the development of successful interventions. For example, many trauma theories of PTSD (Foa et al., 2005) have their origins in information processing theory, which was developed not to understand the impact of trauma on mental health, but originally to describe and explain the mental development of children, which has since been expanded to describe and explain cognitive processes across a wide range of age groups and conditions (Proctor & Vu, 2006). Thus, what many consider to be basic or foundational science in the areas of mental health is actually epidemiological or etiological and therefore these topics will be discussed under those research areas.

### Epidemiology

Epidemiology efforts involve population level descriptive studies of the patterns and causes of health and disease conditions aimed to identify the nature of the risk factors and frequency of chronic effects. Within the military Psychological Health Research Continuum, the focus has been on identifying the impact of combat and deployment on the mental health status of the service member, including risk and resilience factors. Such efforts necessarily require the development or maintenance of databases that allow for the long-term monitoring of the mental health status of combat veterans. Thus, both general and specific databases are needed to achieve these goals.

Although the DoD has funded a number of epidemiological studies to address the mental health issues related to deployment and combat, the DoD's flagship epidemiology study (akin to the Framingham study) is the Millennium Cohort (MILCOHORT) Study, which began in 2000 and is scheduled to run until 2067.

The MILCOHORT is the largest prospective health study in US military history (Ryan et al., 2007) comprising more than 200,000 service members and veterans. Furthermore, it is one of the few medical research efforts that began prior to 9/11, so unbiased baseline rates can be established for many mental and physical health conditions. Findings from the MILCOHORT have provided important information about the onset of new PTSD following deployment and combat, estimated to be between 7.6–8.7% compared to 1.4–3.0% for non-combat or non-deployers (Smith et al., 2008); onset of new depression following combat, estimated to be 5.7% for men and 15.7% for women compared to 3.9 and 7.7% for men and women, respectively, who have never deployed (Wells et al., 2010); and the lack of association of suicides in military personnel deployed to either Iraq or Afghanistan (LeardMann, Powell, et al., 2013) among others.

Another important epidemiological surveillance study of service members’ mental health is the large-scale, 5-year, Land Combat Study initiated in 2003. This study involves anonymous cross-sectional and longitudinal surveys of mental health status of service members from combat infantry brigades at different points in the deployment cycle. Findings from this effort indicated that 12–13% of service members surveyed 3–4 months post deployment met screening definition of PTSD, compared to 5% at baseline (Hoge et al., 2004). Other findings from the Land Combat Study documented that psychological stigma was two to three times as high for those screening positive for a mental health care problem compared to those who didn't screen positive.

Interestingly, the PTSD rates of US service members deployed to Afghanistan appear to be significantly higher compared to other nations such as the United Kingdom and Canada when their service members were deployed to the same location (Hotopf et al., 2006). However, in a joint UK–US study, when key variables such as age, marital status, and combat experiences were adjusted, no significant differences in PTSD rates between UK forces and US forces who deployed to Afghanistan were found (Sundin et al., 2014). PTSD rates for UK forces were 3.9% compared to 3.6% for US forces. Interestingly, and somewhat unexpectedly, alcohol use and aggression were higher for UK personnel than for the US personnel. More than 34% of UK personnel screened positive for alcohol use on the AUDIT compared to just 8.4% for US personnel, with aggression being twice as high in the UK personnel compared to US personnel (17.3 vs. 7.7%).

Another important epidemiology study currently underway is the Army STARRS (Study to Assess Risk and Resilience in Servicemembers) (Schoenbaum et al., 2013). This study is designed to identify the risk and resilience factors associated with the Army suicide rates, which have increased over the past 8 years at an alarming rate (Castro & Kintzle, 2014), especially for the army and the Marines. Suicide rates within the military are now on par with the suicide rates seen in the civilian population, and suicide attempts in the military are more likely to result in death than in the general population (Anestis & Bryan, 2013). For example the rates of suicide for the army have doubled, increasing from 11.1 per 100,000 in 2008 to 22.1 per 100,000 in 2012. There has been much speculation and debate as to the causes of these astonishing increases in suicide rates, ranging from an overall increase in the stress on the force because of a decade of wars, to multiple military deployments, to the stressors of combat, to the lowering of accession standards, among others, including mild traumatic brain injury resulting from concussions. There is no debate that: 1) the stress on the force over the past decade has increased at unprecedented levels; 2) suicide rates are increasing for both deployed service members and for service members that have never deployed; 3) high combat exposure results in significant increases in mental health problems such as depression, anxiety, and PTSD; and 4) mental health problems associated with combat are a significant risk factor for death by suicide. Thus, it is likely the increases in military suicides can be attributed to both an increase of stress on the force and to high combat exposure, possibly mediated through guilt and second-guessing (Bryan, Ray-Sannerud, Morrow, & Etienne, 2013), which leads to severe emotional distress (Bryan, Rudd, & Wertenberger, 2013), that results in an increase in combat-related mental health problems.

The final large, epidemiological effort that must be mentioned is the annual mental health advisory teams (MHATs) which began in 2003 and are designed to assess the mental health of US service members in combat environments, including an evaluation of the in-theatre behavioral healthcare delivery system. Although a detailed report of these teams has been described elsewhere (Bliese, Thomas, McGurk, McBride, & Castro, 2011), it is important to note here that this was the first time in any nation's history that the mental health status of a force was assessed while there were still ongoing combat operations. Other nations have since replicated the US's MHAT approach (Mulligan et al., 2010). Key findings from the MHATs include: 1) deployed soldiers and Marines experience high rates of combat, which are related to increases in PTSD rates (see also Maguen et al., 2013 among others); 2) longer and multiple deployments lead to increases in PTSD, depression, and anxiety rates if service members do not have adequate recovery time between deployments, and 3) combat frequency and mental health problems are associated with ethical mistreatment of non-combatants (see Castro & McGurk, 2007).

### Etiology

Etiological studies are concerned with determining the biological, psychosocial, and environmental causes of a mental health disorder. Critical work funded in this domain includes the characterization of the PTSD disease processes and the underlying neurobiological mechanisms. The objective is to develop a validated model, including risk and resilience factors, symptom onset, recovery, and disorder trajectories. Understanding the interplay between PTSD and other comorbid disorders is also a key focus.

Not surprisingly, adverse childhoods have been shown to be related to depression and PTSD following combat exposure (Cabrera, Hoge, Bliese, Messer, & Castro, 2007); and childhood physical and sexual abuse have been associated with suicide risk for military veterans (Bryan, McNaughton-Cassill, Osman, & Hernandez, 2013). Similarly, military sexual trauma, an often unreported trauma for both men and women, can and does occur during military deployments (LeardMann, Pietrucha, et al., 2013). Military personnel who are especially vulnerable to being sexually assaulted are those who have been previously sexually assaulted prior to entering the military, and military service members sexually assaulted are more likely to suffer from PTSD and other comorbidities such as depression, anger, eating disorders, and suicidality (see Maguen et al., 2012).

The United States has made an enormous investment in biomarker research for PTSD (and to a much lesser extent in suicide research) (see for example Ursano et al., in press). The theoretical plausibility and the practical use of biomarkers for the assessment and treatment of PTSD are convincing (Yehuda, Neylan, Flory, & McFarlane, 2013). DNA methylation (Rusiecki et al., 2012) and glucocorticoid receptor methylation (Yehuda et al., in press) have shown early promise in identifying combat veterans with or without PTSD. Indeed, the glucocorticoid receptor gene, NR3C1, has been shown to predict treatment outcome of combat veterans with PTSD (Yehuda et al., 2013). Likewise, inflammatory proteins have been shown to be associated with PTSD in Marines returning from a combat deployment (Eraly et al., 2014). It is widely anticipated that ongoing and future biomarker research will yield biomarkers that will be useful in the diagnosis and treatment of combat-related PTSD.

### Prevention and screening

Prevention efforts include population, selective, and indicated preventions. Prevention efforts include the development and validation of effective mental health and education training of service members, as well as validated leader training. Risk prevention, risk reduction, and resilience building efforts also represent important prevention efforts. Research aimed at reducing psychological stigma and barriers to seeking treatment are also undertaken. Screening might be viewed as separate from prevention because screening typically involves the identification of the early onset of mental health symptoms or mental health symptoms that have not been detected, yet requiring intervention. Key efforts include the validation of assessment tools and measures, as well as interview screening protocols. In addition to the development and validation of self-report screening procedures to detect mental health issues, the development of “objective” mental health screens is also an important area of research.

Although many countries conduct mental health training to reduce the effects of combat on the mental health status of service members, the only mental health training program for military personnel that has undergone rigorous scientific validation is Battlemind training. The United States was the first country to propose that mental health training before, during, and after deployment could attenuate the adverse effects of combat (McKibben, Britt, Hoge, & Castro, 2009). In a series of studies involving group randomized trials, the Battlemind mental health training program was shown to significantly reduce the mental health symptoms associated with PTSD, depression, and sleep in combat veterans returning from Afghanistan (Adler, Bliese, McGurk, Hoge, & Castro, 2009; Castro, Adler, McGurk, & Bliese, 2012). Since its introduction over a decade ago, other resilience-based training programs have expanded upon the Battlemind concepts, yet all the while maintaining its core elements (Zamorski, Guest, Bailey, & Garber, 2012).

Leaders too are critical for sustaining the mental health of their subordinates. Castro and McGurk provided compelling evidence that when junior leaders display constructive and positive behaviors; the mental health of their subordinates is superior to the mental health of subordinates whose leaders do not engage in such behaviors (Castro & McGurk, 2007). This finding that leaders can play a pivotal role in sustaining the mental health of their subordinates who have been exposed to high combat has opened up a new area of research that has yet to be fully explored.

Mental health screening is one of the most widely used methods for identifying service members returning from combat who might need help for a mental health issue (Wright, Huffman, Adler, & Castro, 2002). The United States was the first country to employ mental health screening on a wide basis, which began in 1995 for all US personnel returning home from the peacekeeping mission to Bosnia. Benefits of mental health screening include: providing a formal process for the opportunity of early mental health intervention; reducing stigma and barriers to care for mental health issues; providing service members an opportunity to self-identify for a mental health issue that is concerning them; familiarizing service members with the military mental health care system; and helping to change the military culture around mental health care (Wright et al., 2002). Despite inherent issues associated with mental health screening, such as low specificity and sensitivity as well as underestimating true prevalence rates of service members in need of mental health care, mental health screening remains the preferred method for early identification of service members with mental health issues (Milliken, Auchterlonie, & Hoge, 2007).

Service members returning home from combat pose unique challenges for the communities receiving them. One day the service member may be conducting combat operations where they are killing the enemy, and the next day they are back home with their family and friends. The experience of Vietnam veterans is often cited as an example when combat veterans were in the jungle one day and back on Main Street in the US the next. Some believe that this sudden transition from the war zone to home contributed to many of the mental health and other adjustment problems seen in Vietnam veterans. On the flip side, for veterans from World War II, it has been suggested that the slow journey home on troop ships allowed these veterans to “decompress” naturally, implying (erroneously) that World War II veterans did not have any mental health issues related to combat.

The United States showed that “decompression” conducted in garrison at the service members’ home station back in the States immediately upon returning home from combat resulted in fewer PTSD symptoms, better sleep, and a healthier transition 4 months later compared to service members who didn't experience garrison-based decompression (see Adler et al., 2009; Castro et al., 2012). Other countries, however, set up decompression sites at third locations for returning combat veterans, arguing that in order for decompression to be effective that it must occur at a site away from family and friends, without external distractions, and be limited to unit members on the combat deployment (Castro, Greenberg, & Vigneulle, 2009). Unfortunately, there is no evidence demonstrating that a third location is necessary for decompression to be effective in preventing combat-related mental health problems. Indeed, in the only rigorous study undertaken, TLD, did not reduce the mental health burden associated with combat (ADF Report, 2012), suggesting that excluding family and friends from the process is not necessary, nor necessarily ideal. In a quasi-experimental study conducted by the United Kingdom, TLD was not effective for those service members in the greatest need of psychological support (Jones et al., 2013).

The research into the effectiveness of psychological debriefings in the prevention of combat-related mental health issues should be mentioned because the use of psychological debriefings remains controversial (Wessely & Deahl, 2003). However, systematic reviews of psychological debriefings (Rose, Bisson, Churchill, & Wessely, 2002) that have been conducted to determine whether it is effective in preventing or ameliorating the adverse behavioral or mental health effects resulting from trauma have included studies involving victims of trauma, who were physically wounded, such as burn and rape victims, as well as victims of motor vehicle accidents, despite the developers of psychological debriefing clearly stating that such individuals should not undergo such debriefing procedures (Everly & Mitchell, 1997). In a large group randomized trial of combat veterans returning from a year-long deployment to Iraq, psychological (Battlemind) debriefing reduced the psychological symptoms associated with PTSD down to 4 months after returning home (Adler et al., 2009). Thus, the use of psychological debriefings, at least with a military context, appears to provide a useful early intervention tool for lessening the impact of combat and deployment stress on the mental health and wellbeing on service members.

### Treatment

Treatment research is aimed at symptom amelioration that covers the different stages of illness. Key treatment modalities include psychotherapies and medications. Treatments that are new or involve repurposed psychotherapies, medications, and combination treatments are also of importance. Treatments that are brief, yet still effective are preferred. Methods for guiding personalized treatments are also highly desirable. Complementary and integrative adjunctive therapies are important. Telemedicine and technology enhanced psychotherapies, which improve treatment effectiveness are also a focus. Treatment research that advances the scientific evidence to improve clinical practice guidelines is highly desirable. Finally, biomarkers that detect the effectiveness of treatment therapies are sought.

The treatment of mental health issues in the military generally follows the same clinical practices for the treatment of civilian mental health issues. Adler and Castro (2013), however, have argued that the treatment of combat-related PTSD should differ from the treatment of non-combat-related PTSD because of significant differences in the nature of the trauma(s), the symptom manifestations and intensity, and the time course of the disorder. Support for this position has indeed been reported. A critical issue is whether approaches to treating other combat-related mental and behavioral health issues will also have be modified and adapted for the military occupation.

When discussing treatments for PTSD, one should appreciate that prior to 9/11 neither of two dominant psychotherapies used to treat PTSD [prolonged exposure therapy (PE) or cognitive processing therapy (CPT)] had ever been shown to be effective in treating combat-related PTSD in either military veterans or in actively serving combat veterans (Foa, Hembree, & Rothbaum, 2007). Thus, a top research priority was to confirm that the current psychotherapies for treating assault-related PTSD would also be effective in treating combat-related PTSD, and whether adjustments to the treatment protocols would be needed. Thus, several major clinical trials were commissioned to assess the effectiveness of PE and CPT alone or in combination with drugs approved for treating PTSD in combat veterans suffering from PTSD who were still serving on active duty. Fortunately, PE has been shown to be effective in reducing PTSD symptoms in veterans from the Vietnam War, the first Gulf War, and from the wars in Iraq and Afghanistan (Yoder et al., 2012). Large clinical trials assessing the effectiveness of PE in active duty service members is currently underway (Peterson, Luethcke, Borah, Borah, & Young-McCaughan, 2011).

There have been two drugs approved by the Federal Drug Administration (FDA) for the treatment of PTSD, Zoloft and Paxil. However, neither of these drugs has ever been tested for their effectiveness in treating combat-related PTSD. Both drugs were tested and approved by the FDA for treating non-combat-related PTSD, usually associated with rape or sexual assault. Clinical trials are currently underway to assess the effectiveness of these drugs in treating combat-related PTSD.

In addition to psychotherapies and drugs to treat combat-related PTSD, the United States has also explored other novel or innovative approaches for either treating combat-related PTSD or interventions to be used as adjuncts for existing treatments. For instance, transcranial magnetic stimulation has been shown in numerous studies to be an effective treatment for PTSD (Karsen, Watts, & Holtzheimer, 2014). In a small pilot study, emotional freedom technique has also been shown to be effective in ameliorating the symptoms associated with PTSD (Church et al., 2013). Furthermore, mindfulness based stress reduction therapy has proved promising in reducing PTSD, depression, and other anxiety-related disorders (Arch et al., 2013). Most recently, ketamine has been shown to be efficacious as a treatment for chronic PTSD (Feder et al., 2014).

Finally, prazosin has been shown to be an effective adjunct for the treatment of PTSD-associated nightmares (Koola, Varghese, & Fawcett, 2014; Raskind et al., 2013).

Advanced technologies and telemedicine have been used in a variety of innovative ways to improve the delivery, assessment, and care of evidence-based therapies to service members and veterans. Video teleconferences have been used to provide behavioral health care to military personnel in isolated, rural areas where behavioral health care providers are not readily available, including using video teleconference technology to support military personnel on deployments or during prolonged exercises (Hill et al., 2004). Indeed, video teleconference procedures have been shown to be effective in delivering CPT (Morland et al., 2014) to combat veterans with PTSD. Smartphone apps have also been used in the management of PTSD symptoms and reactions, such as sleep and anxiety reactions (Kuhn et al., 2014). Computer simulation games have successfully been used as an adjunct for the treatment of alcohol use disorder by allowing veterans with alcohol use disorders to practice relapse preventions skills (Verduin, LaRowe, Myrick, Cannon-Bowers, & Bowers, 2013).

Finally, a number of efforts have been initiated to demonstrate the value of VR as an adjunct to PE for treating combat veterans with PTSD (Rizzo et al., 2010). To date, all reported efforts utilizing VR to assist in the treatment of combat-related PTSD have involved case studies, open trials, with no control groups or randomization or pilot studies (e.g., Reger et al., 2011). Large RCT studies are currently underway to fill this important scientific gap.

### Follow-up care

Follow-up care is focused on assessing the length and durability of treatments, long-term consequences of treatment, rehabilitation, and relapse prevention. Also of interest is the development and validation of return-to-duty standards following a mental health illness or disease. Follow-up care research includes long-term recovery tracking and systems of care. The development of recovery protocols and tools for conducting periodic assessment or re-screening is considered follow-up care. Models that ensure the continuity of care are also developed under this research line, as is the development and validation of collaborative case management.

Although follow-up care research represents a critical area of research, research to date has not been encouraging. For instance, telephone monitoring and support of veterans after discharge from a residential PTSD treatment program did not show improved mental or behavioral health outcomes compared to veterans who not receive follow-up care management (Rosen et al., 2013). In a recently completed review and meta-analysis of remission from PTSD without specific treatment, involving 42 studies with 81,642 participants, it was found that the remission rate was 51.7% 5 months following the trauma compared to 36.9% remission rate beyond 5 months after the trauma, indicating that early treatment is essential for improved prognosis (Morina, Wicherts, Lobbrecht, & Priebe, 2014). Another effort that is underway is to determine if suicide mortality can be reduced following 2-year post discharge following inpatient hospitalization for suicidality through the use of caring emails (Luxton et al., 2014).

### Services research

Services research is broad. Service research is focused on system of care improvements, improving access to care, including understanding mental health service utilization factors; enhancing the delivery of healthcare services, and improving treatment adherence. Services research is also aimed at improving access quality and outcomes of care through improved coordination and consistency of clinical treatment. Maintaining efficacy and fidelity in treatment and care systems, developing effective methods for disseminating best practice information, increasing the adoption of best practices by providers, and developing valid training tools for service, VA, and community providers also fall within the domain of services research.

Efforts to develop collaborative care models to improve primary care management of veterans with PTSD have not been successful (Schnurr et al., 2013). Indeed, those veterans who received telephone management through a coordinated system of care involving a case manager, primary care provider, and a psychiatrist were no different than those veterans who received usual care in symptoms associated with PTSD or in functionality. Instead, those veterans who received coordinated care were more likely to have had a mental health visit and to have received antidepressant medications, suggesting that neither of these additional mental health interventions resulted in any appreciable improvement.

Service members fail to receive mental health care due to psychological stigma and various organizational barriers (Hoge et al., 2004). Numerous research efforts have been initiated to increase the likelihood of service members receiving mental health care by either reducing the stigma and barriers to seeking care or by increasing the motivation of service members to seek care. Initial results using brief motivational feedback have shown that veteran patients with comorbid substance dependence and psychiatric disorder were more likely to enter outpatient treatment groups compared to those veterans who did not receive brief motivational feedback (Lozano, LaRowe, Smith, Tuerk, & Rotzsch, 2013). Likewise, combat veterans who indicated a readiness for change at intake were more likely to utilize outpatient mental care resources (Jakupcak et al., 2013).

The VA embarked on an organizational-wide dissemination program in 2008 to train behavioral health care providers in PE and CPT to improve the delivery of evidence-based psychotherapies to veterans with PTSD (Cook et al., 2013). Despite these noble attempts to disseminate evidence-based care, behavioral health care providers still report wide variation in the adoption of these two treatments for PTSD, ranging from no adoption to nearly full adoption. Furthermore, as last as 2010, only 27% of patients received any psychotherapy, with new cases of PTSD receiving no psychotherapy or a low-intensity amount of psychotherapy (Mott, Hundt, Sansgiry, Mignogna, & Cully, 2014).

## Special populations

### Military families

For the service member, leaving the combat zone marks the beginning of the transition home, with the objective to smoothly reunite with their families, to put the stresses of combat behind them, and prepare to deploy again if needed. Just the same, family members have struggled and will struggle with this reunion (Hazle, Wilcox, & Hassan, 2012). Military families also have unique challenges (Riviere & Merrill, 2011). For example, while a service member is deployed to a combat zone, their family often worries about the health and safety of the service member (Pincus, House, Christensen, & Adler, 2005). Families of deployed service members have always been expected to be encouraging and protective, but their own needs and reactions to deployment have often been understudied or ignored, especially by civilian institutions. By and large, military children and families are largely resilient, characterized by an ability to adapt, take advantage of adversity, and change to learn new skills. On the other hand, not all families display these qualities of durability. “Secondary” PTSD has now been identified as a serious and real threat to spouses, children, and others who must adapt to the disordered behavior of a combat veteran family member (Figley, 1998). Under circumstances in which a returning service member cannot sleep, turns day into night, reacts with unpredictable hostility to daily events, cannot maintain work, and cannot focus, every family member is thrown of balance.

### National Guard and reserves

The National Guard and reserves are also a special group affected by this war. They comprised at any given time during the war between 26 and 40% of the forces deployed to Iraq and Afghanistan. Reservists are in fact twice as likely to demonstrate need for mental health services following deployment, based on screening criteria used by the military (Castaneda et al., 2009). Several factors are thought to contribute to these comparatively poor outcomes. Most reservists live far from military facilities and therefore have little identity as a military family. They lack access to the supportive services and network found at or in military installations (Castaneda et al., 2008).

## Future psychological health research needs for combat veterans

The diagnosis of PTSD has recently been changed (see APA, 2014). The definition of a traumatic event has been changed; the number of symptom clusters has been increased from three to four; and new symptoms, behaviors, and reactions have been added, including persistent negative trauma-related emotions, persistent distorted blame of self and others, risky or reckless behaviors, among others. To a large extent these changes were purported to be motivated by the desire to provide better care for combat veterans who were exposed to the trauma of war. However, the scientific evidence to merit these changes is questionable, and likely to result in confusion around the diagnosis of PTSD, as well as erroneously attributing symptoms to behavior (see Harpaz-Rotem, Tsai, Pietrzak, & Hoff, 2014; Zoellner, Bedard,-Gilligan, Jun, Marks, & Garcia, 2013).

Moreover, the words “combat,” “veteran,” or “military” do not appear anywhere in the diagnostic description, not even in the section devoted to high risk occupations. The scientific evidence for lumping combat-related PTSD, with PTSD related to other forms of trauma such as sexual or physical assaults, and survivors of natural disasters such as hurricanes and floods is questionable (see Adler & Castro, 2013 for a detailed discussion of this concept). Veterans of war often describe their combat and deployment experiences as the most exciting time of their life, something they would be willing do all over again. Such statements are made even by combat veterans who are suffering from PTSD! Yet, neither rape victims nor survivors of natural disasters ever describe their trauma experiences this way, although some individuals in disaster settings might find excitement in such situations that was unknown to them before. Most certainly we are describing two different populations. Additional research will be needed to elucidate these differences.

## Conclusion

The demands of conducting warfare in two combat theaters for over a decade, involving multiple deployments, has dramatically affected the psychological health of the US military forces. The last decade has witnessed a cultural shift in the research landscape regarding mental health research for combat veterans. The transition from combat to back home can be challenging. And the transition from military to civilian life, under the best circumstances, can be difficult and is not always negotiated successfully (Hassan & Flynn, 2012). Given the current and future mental health challenges facing our veterans and their families, military mental health programs and support services have never been more crucial in resolving many of these hardships and in restoring the human potential of our veterans and families. The brave men and women who wear the uniform in combat deserve our deepest gratitude and support.
